# The Early Appearance of Asthma and Its Relationship with Gut Microbiota: A Narrative Review

**DOI:** 10.3390/microorganisms12071471

**Published:** 2024-07-19

**Authors:** Clara Suárez-Martínez, Marina Santaella-Pascual, Genoveva Yagüe-Guirao, Luis García-Marcos, Gaspar Ros, Carmen Martínez-Graciá

**Affiliations:** 1 Biomedical Research Institute of Murcia (IMIB-Arrixaca), 30120 Murcia, Spain; clara.suarez@um.es (C.S.-M.); gyague@um.es (G.Y.-G.); gros@um.es (G.R.);; 2 Food Science and Nutrition Department, Veterinary Faculty, Regional Campus of International Excellence Campus Mare Nostrum, University of Murcia, 30100 Murcia, Spain; 3 Microbiology Service, Virgen de La Arrixaca University Clinical Hospital, Regional Campus of International Excellence Campus Mare Nostrum, University of Murcia, 30120 Murcia, Spain; 4 Pediatric Allergy and Pulmonology Units, Virgen de La Arrixaca University Clinical Hospital, Regional Campus of International Excellence Campus Mare Nostrum, University of Murcia, 30120 Murcia, Spain; 5 Network of Asthma and Adverse and Allergic Reactions (ARADyAL), 28029 Madrid, Spain

**Keywords:** asthma, gut microbiota, gut–lung axis

## Abstract

Asthma is, worldwide, the most frequent non-communicable disease affecting both children and adults, with high morbidity and relatively low mortality, compared to other chronic diseases. In recent decades, the prevalence of asthma has increased in the pediatric population, and, in general, the risk of developing asthma and asthma-like symptoms is higher in children during the first years of life. The “gut–lung axis” concept explains how the gut microbiota influences lung immune function, acting both directly, by stimulating the innate immune system, and indirectly, through the metabolites it generates. Thus, the process of intestinal microbial colonization of the newborn is crucial for his/her future health, and the alterations that might generate dysbiosis during the first 100 days of life are most influential in promoting hypersensitivity diseases. That is why this period is termed the “critical window”. This paper reviews the published evidence on the numerous factors that can act by modifying the profile of the intestinal microbiota of the infant, thereby promoting or inhibiting the risk of asthma later in life. The following factors are specifically addressed in depth here: diet during pregnancy, maternal adherence to a Mediterranean diet, mode of delivery, exposure to antibiotics, and type of infant feeding during the first three months of life.

## 1. Introduction

Asthma is the major non-communicable disease affecting both children and adults, with high morbidity and relatively low mortality compared with other chronic diseases [1,2]. It is a condition with several clinical phenotypes whose principal characteristics include a variable degree of airflow obstruction; bronchial hyper-responsiveness; and airway inflammation that leads to recurrent episodes of wheezing, breathlessness, chest tightness, and coughing, particularly at night or early in the morning [3,4]. Severity ranges from occasional symptoms to disabling persistent symptoms and/or frequent life-threatening exacerbations [5]. However, in children <5 years of age, clinical symptoms of asthma are variable and non-specific [3]. Asthma and allergic diseases such as allergic rhinitis (hay fever), atopic dermatitis (eczema), and immunoglobulin E-mediated food allergy are closely associated, with the likelihood of developing asthma being increased by a personal or family history of allergic disease [5,6]. Several clinical phenotypes of asthma have been defined [4]; some of the most common are allergic asthma, non-allergic asthma, adult-onset (late-onset) asthma, asthma with persistent airflow limitation, and asthma with obesity; allergic asthma and non-allergic asthma are the most easily recognized. Both have similar manifestations of airway hyper-responsiveness and inflammation, mediated by different mechanisms. By definition, allergic asthma is associated with allergen sensitization, hypersensitivity mediated by immunoglobulin E (IgE), and type-2 immune response (Th2). On the other hand, non-allergic asthma is associated with neutrophilic inflammatory response with Type-1 (Th1) and Type-17 (Th17) cytokine profile. Allergic asthma is initiated by mast cell activation in response to allergen-binding IgE receptor and Th2-cells, which can directly induce airway hyper-responsiveness and play an important role in initiating and generating the physiological abnormalities in the asthmatic subjects. This leads to mast cells producing and releasing cytokines and recruiting eosinophils and other inflammatory cells. The activation of these cells triggers the release of pro-inflammatory mediators, among which interleukin 4 (LTC4) has a key role. Mediators such as Histamine, LTC4, and Tryptase produce airways’ smooth muscle contraction [7]. The current therapy for asthma is based on the achievement of several objectives, such as relaxation of the smooth muscle of the airways and prevention and reversal of inflammation [8].

Epidemiologic studies have shown that, over the last few decades, the prevalence of asthma in the pediatric population (0–17 years) has increased at an incidence rate of 1.4% per year, and, overall, the risk of developing asthma is highest in children during the period between birth and four years of age [9,10]. Asthma is estimated to affect approximately 300 million people worldwide, and it is likely that, by 2025, an additional 100 million might be affected [2,11]. Asthma also causes significant healthcare expenditure. Uphoff et al. in 2017 [12] examined variations in the prevalence rates of childhood asthma and wheeze at 4 years of age in different European countries and found that the prevalence of asthma varied from 1.72% in Germany to 13.48% in England, and that of wheeze from 9.82% in Greece to 55.37% in Spain. In the International Study of Asthma and Allergies in Childhood (ISAAC), the overall prevalence of wheeze was estimated to be 11.7% for 6–7-year-old children [13]. 

The hygiene hypothesis and the “lost microbe” hypothesis assume that the loss of specific bacteria from the modern-day human microbiota is caused by to our increasingly hygienic lifestyle, C-section deliveries, and excessive use of antibiotics [14,15]. Due to the dramatic changes in lifestyle in the last century, the human microbiota suffers from a loss of diversity, and that loss is one of the causes of the recent increase in the prevalence of asthma [10,16]. On the other hand, and dealing with a broader range of conditions, the Developmental Origins Hypothesis for Health and Disease (DOHaD), or “perinatal programming”, proposes that nutritional and other environmental stimuli can “program” developmental, metabolic, and immune pathways during critical periods of prenatal and postnatal development and subsequently induce long-term changes in metabolism and susceptibility to chronic diseases, including asthma [16,17]. Immune system development begins in utero and continues during the first years of life. A Th2 response is promoted in the fetal immune system by the maternal environment during pregnancy, which is thought to protect the fetus from immunologic rejection by the mother, and it is after birth when the transition to a non-allergic Th1 phenotype occurs. If, during the early postnatal life, this transition is delayed or damaged, there is an increased risk of atopic disease, including atopic dermatitis, allergic rhinitis, allergic conjunctivitis, anaphylaxis, and asthma [16]. Gut microbes have been shown to induce regulatory T cells that help guide the host’s Th1/Th2 balance, and it has been shown that the recognition of microbiota-derived peptides by mucosal receptors enhances systemic innate immunity [18]. Also, two studies in germ-free mice show how, during the postpartum stage, the immune system produces an immune response directed by Th2 cells, but after the restoration of the intestinal microbiota, there is a shift towards a normal immune phenotype, dominated by type cells Th1 and Th17 [19]. This suggests that the intestinal microbiota plays a key role in establishing the balance between the Th1 and Th2 phenotypes during the early stages of life [19]. Microbial metabolites, including SCFAs, could influence the development of asthma, since their presence and variety in maternal and infant feeding has been associated—however, inconsistently—with childhood asthma [20]. The growing body of epidemiological and microbiological literature supports the hypothesis that the inception of allergic disease and, by extension, asthma development may lie at least in part in the communities of microbes that exist in the gastrointestinal tract; it is called gut–lung axis [9]. Although the exact mechanisms by which this axis would activate the innate immune system are not wholly known, there is evidence of possible interactions between the gut and lung mucosa [21]. It is likely that this axis is important to maintain normal microbiota and influence the immune response in both compartments and maintain homeostasis [22]. Some factors have been identified as related to atopic disease development in childhood, such as intrauterine exposure, specifically exposure to antibiotics and Mediterranean diet (MD) adherence; early life antibiotic exposure; C-section [23,24,25]; formula feeding; or lack of exposure to pets during pregnancy [26].

This narrative review aims to summarize the latest scientific evidence concerning the impact of factors such as maternal diet during pregnancy, adherence to a Mediterranean diet, mode of delivery, antibiotic exposure, and early infant feeding practices on the neonatal gut microbiota profile. The review intends to elucidate how these factors influence the risk of asthma and asthma-like symptoms in childhood, emphasizing the concept of the “critical window” within the first 100 days of life as crucial for long-term pulmonary health outcomes. Additionally, the review identifies potential gaps in knowledge that warrant further research.

## 2. Methods

A literature search was performed using the electronic databases PubMed/Medline and Scopus with no date limits. The most relevant published studies were identified in an independent way by the authors.

The keywords used were (in combination) asthma, gut–lung axis, gut microbiota, lactation, breast milk, Mediterranean diet, factors, antibiotics, delivery, dietary pattern, antibiotic, early life, caesarean section, and immune system. The inclusion criteria for the review included (i) original studies published in English; (ii) reviews/systematic reviews, meta-analyses, randomized controlled trials/experimental studies, and observational studies (case, cross-sectional, case–control, and cohort reports); (iii) with defined exposure; (iv) gestational diet and type of diet during the first months of life of the offspring; and (v) mode of delivery.

## 3. Gut–Lung Axis: Mouse and Longitudinal Human Studies Define the Early Life Critical Window

Within the scientific community, a new concept has been established: the “gut–lung axis”, which attempts to mechanistically describe how microbes in the gut might influence immune function in the lung [27,28]. As Stiemsma and colleagues reviewed [29], one of the main connections is through interactions of the intestinal microbiota with pattern recognition receptors of the innate immune system. It is well known that Toll-like receptor (TLR) signaling can be stimulated by pathogen-associated molecular patterns (PAMPs) such as lipopolysaccharide (LPS) and peptidoglycan. This stimulation confers the downstream activation of many genes that regulate inflammation and modulates innate immune responses. Dendritic cells (DCs) are also the intermediaries of gut microbiota–immune cell crosstalk in a similar way to the antigen-recognition and IgE-mediated hypersensitivity pathways, since dendritic cells regularly take samples of gut microbes in the intestinal lumen or lymphoid tissues. The detection of intestinal microbiota PAMP by DCs promotes immune tolerance in the intestine, but also produces phenotypic changes in DCs and its migration to the mesenteric lymph node (MLN) to activate and promote the differentiation of distinct effector T helper-cell subset. In the MLN, T cells acquire targeting molecules such as chemokines, which activate the T-cell migration to other parts of the body, including the respiratory tract mucosa. Therefore, it is possible that phenotypic changes occur in DCs due to interactions with specific gut microbes through their corresponding PAMPs, which would produce subsequent effects on lymphocyte priming/homing and, therefore, changes in anti-inflammatory responses in the airways [30,31].

Another area of gut–lung axis research involves microbial-derived metabolites, such as SCFAs. These metabolites are known to modify gene expression through the inhibition of histone deacetylases (HDACs); cytokine and chemokine production; and cell differentiation, proliferation, and apoptosis [20]. SCFAs function as HDACs inhibitors and ligands, which are predominantly agonists of G protein-coupled receptors (GPCRs). The ability of SCFAs to inhibit HDACs generally promotes a tolerogenic, anti-inflammatory cell phenotype necessary for the maintenance of immune homeostasis [32,33]. SCFAs can also modulate T cells, particularly regulatory T cells (Tregs), through HDACs inhibition [29]. Diverse studies, characterizing the ability of specific SCFAs to regulate the quality of the colonic Treg cell pool, have shown that propionate and butyrate induce FoxP3 (a transcription factor which, following exposure to allergens, is critical for the conservation of immune system homeostasis and responsible for the suppression of Th2 responses [34]) in an HDAC-dependent manner, while acetate is less effective [35,36]. Clostridia species are large producers of SCFAs, and their production of butyrate has been associated with the generation of peripheral Tregs in the colon [36]. In a house dust mite (HDM)–mouse model of experimental asthma, both acetate and propionate were capable of reduce cellular infiltration into the airways [37]. In a later study using the same asthma mouse model, maternal intake of acetate was shown to reduce allergic airway disease in the in the mice offspring when adult [38]. In both studies, the authors demonstrated that a reduced severity of allergic airway inflammation in the offspring was associated with an increase in the intake of fermentation of microbiota-accessible carbohydrates (MACs) during the pregnancy period.

Studies in animal models have made significant advances in our understanding of the gut–lung axis, and in many of them, it has been found that the result is time sensitive, which leads us to the concept “early life critical window”. These studies have been based on the identification of large-scale changes in intestinal microbial compositions in asthma- and allergy-induced mice models, and in the manipulation of the intestinal microbiome with antibiotics, increasing the severity of those diseases [39,40,41,42]. Cahenzli et al. [42] demonstrated that an increased microbial diversity in early life is required to regulate IgE production, and it decreased disease severity in a mouse model of antigen-induced oral anaphylaxis. Lyons et al. [39] showed that perinatal exposure to *Bifidobacterium longum* AH1206 induces Treg levels in both infants and adult mice and protects against OVA-induced Th2 sensitization and allergic airway inflammation in adult mice. Moreover, Russell et al. [40,41] reported that perinatal exposure to antibiotics exacerbates lung disease in adult mice. Specifically, the authors showed that perinatal (in utero and up to 21 days after birth) versus strictly prenatal (in utero) vancomycin treatment of ovalbumin (OVA)-challenged mice exacerbated asthma-related immune responses.

**Figure 1:**
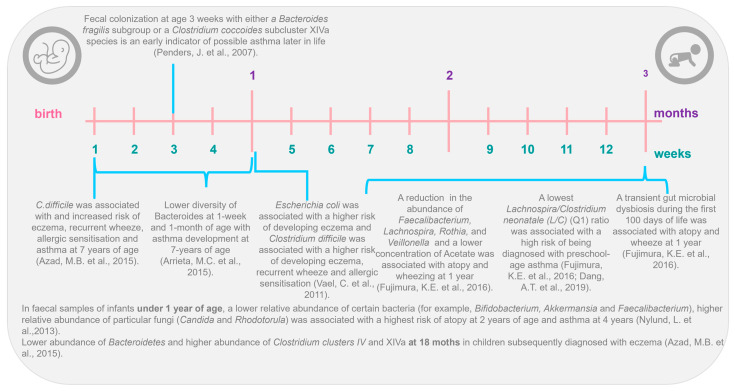
The gut–lung axis: the first 3 months of life as the critical window in early life [41,42,43,44,45,46,47,49,50,53].

The gut–lung axis also is supported by different longitudinal studies (Figure 1), such as in the Canadian Healthy Infant Longitudinal Development (CHILD) cohort, which shows that intestinal microbiota profiles differ between infants who develop and do not develop asthma [43,44,45]. Nylund et al., in 2013 [46], in their prospective study, found a lower abundance of Bacteroidetes and greater abundance of Clostridium clusters IV and XIVa at 18 months in children subsequently diagnosed with eczema. Similar results were founded by Abrahamsson et al. [43], who, using 454-pyrosequencing in feces from a Swedish cohort of 40 infants, associated atopic eczema at 2 years and asthma development at school age with lower diversity of Bacteroidetes at 1 week and 1 month of age. Recent findings from the CHILD cohort study, using next-generation sequencing (NGS), observed that a higher ratio of Enterobacteriaceae to Bacteroidaceae at 3 months of age was predictive of food sensitization at 1 year [47]. Arrieta et al. [44], in a nested case–control study of the same CHILD cohort, examined the gut microbiota of 319 infants and found that children with a high risk of asthma at school age (classified as those with atopy and wheeze at 1 year) exhibited transient gut microbial dysbiosis during the first 100 days of life. Specifically, they identified decreases in the abundances of four bacterial genera, *Lachnospira*, *Veillonella*, *Rothia*, and *Faecalibacterium* in the 3-month fecal microbiota. Other studies have identified shifts in specific bacterial taxa in early life. For example, in the KOALA Birth Cohort, colonization with *Clostridium difficile* at 1 month of age was associated with increased risk of eczema, recurrent wheeze, allergic sensitization, and asthma by 7 years of age [48]. Other studies have shown that colonization with that same pathogen was associated with an increased risk of future wheeze or asthma [49,50]. 

In 2016, Stiemsma et al. [51] found that *Lachnospira* remained low in the 3-month fecal microbiota, while one bacteria species, *Clostridium neonatale*, was increased in infants classified as asthmatics at 4 years of life, at this time-point. The authors calculated a ratio of *Lachnospira* to *C. neonatale* (L/C) and showed that children with the lowest L/C ratio (quartile 1) were 15 times more likely to be diagnosed with preschool-age asthma than children in the other L/C quartiles [51]. Interestingly, both in the study by Arrieta et al. [44] and the study by Stiemsma et al. [51], the authors identified the gut microbial changes only in the first 3 months of life, highlighting this time frame as the early life critical window during which gut microbial dysbiosis is most influential in promoting asthma and atopic disease in humans. With this information, it is more and more evident that gut microbial dysbiosis is associated with human atopic diseases and that it is characterized by taxa-specific shifts in abundance at the family, genus, and even species level and not by global changes to the composition of the intestinal microbiota [44,45,51]. Also, with all the research carried out, it has been determined that the “critical window” to identify these intestinal microbial changes in humans could only cover the first 100 days of the newborn’s life. Furthermore, several factors can modify the profile of the intestinal microbiota [52] and therefore cause dysbiosis: only some of them might contribute to the increasing prevalence of asthma [53]. In the next section, we focus on some of those factors that can modify the intestinal microbiota profile and therefore enhance or inhibit the risk of asthma during later stages of life.

## 4. Factors Related to the Development of Asthma and Allergy during Early Life

### 4.1. Maternal Diet during the Pregnancy 

The relationship between a mother’s diet during pregnancy and the child’s subsequent risk of developing asthma or atopy has become a topic of growing investigation [54] (Table 1). In the review by Amati et al. [55], MD was defined as a “diet characterized by a high intake of fruits, vegetables, whole grain cereals and bread, legumes, fish, and nuts; low-to-moderate consumption of dairy products and eggs, and limited amounts of red meat. It is low in saturated fats and high in antioxidants, fibre, and mono and polyunsaturated fatty acids (PUFAs) mainly derived from extra virgin olive oil and oily fish (n-3 PUFAs)”. MD is known to have many beneficial effects against diseases, as shown by high-quality studies and meta-analyses [56,57,58], making it the most widely studied and evidence-based dietary approach to healthy eating and disease prevention [59,60,61]. For example, in 2008, Chatzi et al. [62] found that adherence to an MD during pregnancy was associated with protection from persistent wheeze and atopy in children. Nurmatov et al. [57] concluded that adherence to MD was protective for persistent wheeze and atopy; and García-Marcos et al. [56], in another systematic review, assessed if adherence to MD was associated with the prevalence of “current wheeze”, “current severe wheeze”, or “asthma ever”, concluding that adherence to MD tended to be associated with lower occurrence of the three respiratory outcomes. Castro Rodriguez et al. [63] also concluded in their review that adherence to MD by the mother during pregnancy showed some protective effect on asthma and/or wheeze symptoms in the offspring, but only during the first year of life. Yin Zhang et al., in 2019 [58], in a systematic review and meta-analysis of observational studies concluded that a high adherence to MD during pregnancy may have short-term effects on wheeze in the first 12 months. These results coincide with those found by other authors that observed a protective effect of MD on wheezing, current allergic rhinitis, and atopy, but none on eczema.

Focusing on the different food groups that make up the MD, a high maternal intake of dairy products during pregnancy may reduce the risk of wheeze in the first year of life, while a high intake of meat may increase it [62]. Miyake et al., in 2010 [64], investigated the association between maternal intake of vegetables, fruits, and selected antioxidants during pregnancy and the risk of wheeze and eczema in the offspring at 16–24 months of age. Higher maternal consumption of green and yellow vegetables, citrus fruit, and beta-carotene may be protective for the development of eczema in the offspring; also, higher maternal vitamin E intake may reduce the risk of infantile wheeze. In a study carried out by Baïz et al. in 2019 [65], the authors observed a significant inverse association between the consumption of cooked green vegetable before pregnancy and childhood asthma; and, for the first time, they noted a significant positive association between meat intake during pregnancy and an increased risk of wheezing, allergic rhinitis, and atopic dermatitis. In 2022, Delvert et al. [66] related the consumption of different food groups during pregnancy to the development of allergic and pulmonary diseases in childhood and concluded that maternal diet quality during pregnancy was not associated with allergic and respiratory multimorbidity groups up to 8 years. However, an infrequent consumption of legumes was associated with a higher risk of suffering from multiple allergic and respiratory diseases (food allergy, eczema, wheezing, asthma, and rhinitis) during childhood [66].

If we focus more specifically on macronutrients and micronutrients that make up a diet, we see that there is current evidence of an association between the intake of certain nutrients during pregnancy and asthma, wheeze, or atopic conditions in childhood. 

One of the micronutrients present in MD is long-chain polyunsaturated fatty acids (LC-PUFAs), which are fatty acids with two or more unsaturated double bonds and a carbon chain length of at least 20 [67,68]. LC-PUFAs typically include omega-3 (n-3), omega-6 (n-6), and omega-9 (n-9) polyunsaturated fatty acids (PUFAs). Among them, n-3 and n-6 PUFAs have important physiological functions and are the ones usually included in studies of asthma prevention [69,70]. The main n-6 LC-PUFA is arachidonic acid (AA), while the main n-3 LC-PUFAs are eicosapentaenoic acid (EPA) and docosahexaenoic acid (DHA). AA is obtained from meat and eggs, while EPA and DHA are picked up from fish, especially fatty (or “oily”) fish, and from supplements (“fish oils”). AA and DHA are the main LC-PUFAs in human breast milk [71] and have been identified to have important roles in the development of the infant visual and cognitive systems [72]. The changes in the n-3/n-6 LC-PUFA ratio in the diet, especially the increased consumption of n-6 LC-PUFA and the decreased consumption of n-3 LC-PUFA, are linked with an increased risk of asthma or wheeze [73,74,75]. It has been hypothesized that the intake of higher n-3 and lower n-6 PUFAs contributes to a decrease in asthma inception [5,76,77].

On early immune development, especially in the context of risk of allergies and asthma, there has been much interest in the role of LC-PUFAs in general, and of n-3 LC-PUFAs in particular. LC-PUFAs have been reported to regulate the function of many immune cell types, including neutrophils, monocytes, macrophages, dendritic cells, T cells, and B cells [78]. The best-known function of LC-PUFAs in immune function, including inflammation, is their role as substrates for the generation of bioactive lipid mediators [79,80]. These mediators are collectively termed “specialized pro-resolving mediators” (SPMs) since they resolve (“turn off”) ongoing inflammation [81,82,83]. Several SPMs also promote aspects of innate immunity, including phagocytosis of bacteria and cellular debris [84]. SPMs include resolvins, protectins (also known as neuroprotectins), and maresins. EPA and DHA promote E- and D-series resolvins, respectively, while both protectins and maresins are produced from DHA [81,82,83,84,85].

When the biological actions of the different lipid mediators formed from AA, EPA, and DHA are considered (LC-PUFAs), a balanced supply of precursors would be important to achieve “optimal” immune cell membrane contents and would contribute to an immune system that helped in protection against pathogens, whilst avoiding the adverse effects of exaggerated inflammation [67].

Benefits of higher prenatal intake of n-3 PUFAs on childhood asthma have been demonstrated in some observational and interventional studies. However, positive and negative findings are mixed. Similarly, while some studies link increased prenatal n-6 PUFA intake to increased childhood asthma risk, other do not [67,77,86,87,88,89].

Thus, it can be concluded that there is no clear relationship between prenatal intake of LC-PUFA and the prevalence of asthma symptoms in the offspring [88]. Moreover, some other factors must be considered, such as the source of PUFA: the origin of the fatty acids is dietary, and there may be other factors in the diet that may act as confounders. This is the case of fish, which provides nutrients of relevance to inflammation and allergic pathways other than n-3 LC-PUFAs, including vitamin D, selenium, and zinc [67]. Dosage and supplementation time; susceptibility of LC-PUFA; active maternal asthma; smoking during pregnancy; and differences in ethnicity, sex, and environment, among other factors, are factors to consider to better understand the efficacy of n-3 LC-PUFA supplementation during pregnancy for protection against asthma/wheeze or other relevant allergic diseases.

In relation to micronutrients, Beckhaus and collages, in 2015 [90], in a systematic review and meta-analysis including 120 titles, abstracts, and citations, as well as 32 studies (29 cohorts), concluded that the current evidence suggests a protective effect of maternal intake of each of the three elements (vitamin D, vitamin E, and zinc) against childhood wheeze. Their effect on asthma and other atopic conditions is inconclusive. More recently, the SEATON birth cohort (Study of Eczema and Asthma Tcohort Observe effects of Nutrition) [91] found that lower maternal vitamin D and E intakes during pregnancy are associated with increased risk of wheezing and asthma diagnosis in the first 10 years of life, but not after puberty. Other studies have concluded that low maternal vitamin D intake from foods during pregnancy may be negatively associated with the risk of asthma in childhood [92]. Vitamin C may also play an important role in the prevention of wheeze and asthma since it was observed that vitamin C supplementation during pregnancy was associated with a small but significantly increased offspring’s airway function at 3 and 12 months of age [93,94].

Folic acid is an essential vitamin during pregnancy, and since the early 1990s, maternal folic acid supplementation has been recommended prior to and during the first trimester of pregnancy. It belongs to the group of B vitamins, and it contributes to the proper development of the neural tube and plays an important part in the synthesis of nucleic acid and protein by donating methyl groups. Several studies have tried to clarify the role of the intake of folic acid during pregnancy on the early onset of asthma in the offspring [95,96]. For example, in a retrospective cohort study of 104,428 children, born in 1996–2005, and their mothers, the authors concluded that children born to women who consumed folic acid supplementation at any time during pregnancy have an increased risk of asthma as compared with children born to women with no folic acid supplementation [96]. No association was seen in children born to women exposed to folic acid supplementation after the first trimester [96]. In another large prospective cohort of children born between 2002 and 2006 from the Norwegian Mother and Child Cohort Study, pregnant women taking supplemental folic acid at/or above the recommended dose (400 µg/d), combined with a diet rich in folate, reach a total folate intake level which was associated with a slightly increased risk of asthma in children [95]. But the findings are controversial, as other studies suggest that maternal folic acid intake during pregnancy is not associated with asthma development in the offspring [97], or even that early folic acid or prenatal supplementation of atopic women may be important to prevent wheeze among the offspring [98]. A meta-analysis, including a total of 10 studies on maternal folate intake and 5 studies with blood folate levels during pregnancy, concluded that it increased the risk of infant wheezing and that this was similar across geographic regions (Europe and North America) [99]. 

In a more recent meta-analysis by Feng et al. in which twenty-two prospective cohort studies enrolling 130,547 individuals were included, high vitamin D, vitamin E, and zinc intakes in pregnancy were found to be protective factors for wheeze; high β-carotene and magnesium intakes showed protection for eczema; and high vitamin D apparently protected from asthma. Conversely, high folic acid intake could produce excess asthma risk in the offspring [100].

Another important issue to identify macronutrients and micronutrients that may influence the onset of childhood asthma is to understand what is the mechanism that may cause it. For example, as Yong et al. [76] reviewed, traditional natural products such as garlic, curcumin, and broccoli could modify the epigenetic programming through DNA methylation and histone acetylation and, in this way, prevent allergic disease in the perinatal period. In addition, supplementation with dietary methyl donors, such as folic acid, might increase DNA CG methylation by suppressing the expression of immune genes, such as runt-related transcription factor 3 (Runx3), and promoting the development of asthma. Runx3 is a specific gene that modulates Th1/Th2 balance and, hence, the production of interleukins, which induce inflammatory responses. [76]. As for vitamin D, Sandhu et al. [101] concluded that it moderates Th1, Th2, and Treg cells, enhancing the release of interleukin-10 (IL-10). At the same time, vitamin D plays an essential role in cellular metabolism and differentiation via its nuclear receptor (VDR) that may enhance epigenetic regulatory enzymes. In the case of vitamin E, during the oxidation of fats, it stops the production of reactive oxygen species that are formed in that process, thus being considered an antioxidant compound. This antioxidant action may be the key for the mechanism of preventing asthma, since the antioxidant effect is known to change the Th1/Th2 balance towards Th1 [76].

**Table 1:** The effects of diet and macro- and micronutrient exposure during gestation, and the risk of developing asthma.

References		Year		Type of Study		Study Population		Objective		Methods		Summary of Major Findings
Delvert et al. [66]		2023		Cohort study		1316 mother–child pairs from the EDEN cohort		To investigate the associations between maternal-diet quality during pregnancy and allergic and respiratory multimorbidity clusters in children.		Dietary assessment through food-based and nutrient-based scores; identification of multimorbidity clusters using Latent Class Analysis; multinomial logistic regressions to assess associations.		Four clusters identified: “asymptomatic”, “asthma only”, “allergies without asthma”, and “multi-allergic”. No general association with maternal diet-quality scores, but a higher risk of “multi-allergic” cluster for children whose mothers consumed legumes once a month or less (OR = 1.60). Suggests potential benefits of legume consumption during pregnancy for preventing allergic diseases.
Komulainen et al. [89]		2023		Randomized, double-blind, placebo-controlled trial		439 pregnant women and their children up to 2 years old		To investigate the effects of fish oil and/or probiotics during pregnancy on the incidence of allergic diseases in children.		Participants were randomized into four groups: fish oil + placebo, probiotics + placebo, fish oil + probiotics, and placebo + placebo. They received daily supplements from early pregnancy until 6 months postpartum. Data on allergic diseases in children were collected at 12 and 24 months.		No significant differences in the incidence of food allergy, atopic eczema, or atopy among the four groups were found. However, the group receiving probiotics showed a reduced risk of recurrent wheezing at 24 months. While fish oil and/or probiotics did not reduce the overall odds of allergic diseases, probiotics alone might help reduce wheezing, suggesting potential long-term benefits in preventing asthma.
Flom et al. [77]		2021		Cohort study		412 mother–child dyads		To evaluate whether associations between prenatal PUFA intake and childhood asthma are modified by prenatal active maternal asthma or child sex.		Dietary intake of PUFAs was assessed using food-frequency questionnaires. Child asthma outcomes were evaluated prospectively to 4.0 plus or minus 1.7 years. Data were analyzed using logistic regression models, considering maternal asthma status and other covariates.		Higher maternal intake of PUFAs, particularly omega-3 and omega-6 fatty acids, was associated with a reduced risk of asthma in children, but this protective effect was more pronounced in children of mothers without active asthma during pregnancy. The study highlights the potential of maternal diet in influencing child respiratory health, with maternal asthma status being a significant modifier of these associations.
Devereux et al. [91]		2019		Cohort study		1924 children recruited in utero		To investigate if the associations between maternal vitamin D and E intakes during pregnancy and childhood wheeze/asthma outcomes at age 5 and 10 years persist at age 15 years.		Maternal vitamin D and E intakes assessed via food-frequency questionnaire; respiratory questionnaire and healthcare data on asthma treatment collected at age 15; combined data on asthma from ages 1, 2, 5, 10, and 15.		Higher maternal vitamin D and E intakes linked to reduced likelihood of asthma diagnosis in the first 10 years but not beyond puberty, indicating post-natal exposures predominate in the etiology of incident asthma
Baïz et al. [65]		2019		Cohort study		1140 mother–child pairs from the EDEN cohort study in France		To examine the association between maternal diet before and during pregnancy and the risk of asthma and allergic rhinitis in children.		Dietary intake was assessed using food-frequency questionnaires. Health outcomes (asthma, wheezing, and allergic rhinitis) were determined using parental questionnaires at ages 1, 2, and 3 years. Statistical analysis included logistic regression models adjusting for potential confounders.		Higher maternal consumption of eggs and raw and cooked vegetables during pregnancy was associated with a lower risk of asthma, wheezing, and allergic rhinitis in children. The study suggests the immunomodulatory properties of antioxidants, selenium, and vitamin E in these foods may offer protective effects against allergies.
Viljoen et al. [92]		2018		Prospective cohort study		897 mother–child pairs from the Lifeways Cross-Generation Cohort Study, Ireland		To establish whether vegetable, oily fish, and vitamin D intakes during pregnancy are associated with childhood asthma risk over a 10-year period.		Maternal dietary intake during pregnancy: food-frequency questionnaire. Asthma status in children was determined through general practitioner diagnosis at ages 3, 5, and 10 years.		Higher daily vitamin D intake during pregnancy was associated with reduced odds of asthma in offspring. A higher intake of oily fish and vegetables showed a trend towards reduced asthma risk, but the results were not statistically significant.
Parr et al. [95]		2017		Population-based cohort study		Norwegian Mother and Child Cohort Study, involving mother–child pairs		To investigate the association between maternal folate intake during pregnancy and the risk of asthma in children.		Current asthma at age 7 was defined by asthma medications dispensed at least twice in the year (1901 cases; n = 39,846) or by maternal questionnaire report (1624 cases; n = 28,872). Maternal folate intake was assessed with a food-frequency questionnaire validated against plasma folate.		Higher maternal folate intake during pregnancy was associated with a reduced risk of asthma in children at 7 years of age.
Veeranki et al. [96]		2015		Retrospective cohort study		104,428 children born between 1996 and 2005 and their mothers enrolled in Tennessee Medicaid		To investigate the association between the timing of folic acid-containing prescription filling during pregnancy and childhood asthma at ages 4.5–6 years.		Retrospective cohort study categorizing women into exposure groups based on the timing of folic acid-prescription filling; defined asthma using asthma-specific healthcare visits and medication fills; used logistic regression models to adjust for potential confounders.		15% of children had asthma. Children born to women with folic acid-prescription exposures in the first trimester only or first trimester and later had increased odds of asthma (adjusted OR = 1.2, 95% CI = 1.1–1.3). No association was seen for children born to women exposed after the first trimester.
Miyake et al. [64]		2010		Cohort study		763 mother–infant pairs from a Japanese population		To investigate the association between maternal consumption of vegetables, fruits, and antioxidants during pregnancy and the risk of wheeze and eczema in infants.		Dietary intake was assessed using a self-administered diet-history questionnaire. The occurrence of wheeze and eczema in infants was determined using a questionnaire completed by the mothers when the infants were 16-to-24 months old		Higher maternal intake of green and yellow vegetables and citrus fruits during pregnancy was associated with a reduced risk of wheeze in infants. No significant association was found between maternal intake of total vegetables, total fruit, or antioxidants and the risk of eczema in infants.

### 4.2. Mode of Delivery 

As mentioned above, the hygiene hypothesis suggests that the immune system of newborns is polarized towards Th2 cells because of a rise in the caesarean section rate and could diminish initial microbial exposure and, thereby altering Th1/Th2 development and the transition from Th2 to Th1 type that occurs after birth and increasing the risk of developing atopy [102]. To test this hypothesis, different studies performed in the last two decades have examined whether the mode of delivery influences the risk of developing subsequent atopic conditions and asthma because bacterial colonization of the gut in caesarean section delivery infants (CSDIs) differs from that in vaginally delivered infants (VDIs) [49,103,104,105,106,107]. The results have been inconsistent: some studies reported a relatively increased risk of asthma in CSDIs, along with a lower frequency of atopic sensitization and allergy development in VDIs [108], whereas others disputed it [109] (Table 2). 

Jakobsson et al., in 2014 [110], studied the postnatal intestinal colonization pattern in 24 VDIs (n = 15) or via CS (n = 9). The intestinal microbiota was characterized using pyrosequencing of 16S rRNA genes at 1 week and 1, 3, 6, 12, and 24 months after birth. Furthermore, the authors measured venous blood levels of Th1- and Th2-associated chemokines at 6, 12, and 24 months. The results showed that CSDIs had lower diversity in their total microbiota during the first 2 years of life, had lower abundance and diversity of the Bacteroidetes phylum, and were less often colonized with the Bacteroidetes phylum. Also, CSDIs had significantly lower blood levels of the Th1-associated chemokines CXCL10 and CXCL11. Adeyeye et al., in 2018 [111], examined whether CS increased the risk of wheeze or food allergy in early childhood as compared with vaginal delivery and whether these associations were mediated by breastfeeding. The study population was the Upstate KIDS cohort (2008–2010) of 5753 mothers and infants. The authors concluded that emergency CS (n = 1356) was associated with an elevated risk of wheeze (adjusting for pregnancy complications, maternal atopy, gestational age, birth weight, and smoking during pregnancy) and increased risk of food allergy (adjusting for maternal atopy, pre-pregnancy body mass index, smoking during pregnancy, and parity). However, no significant associations were found in any of the results in the case of a planned CS (n = 1565 infants).

Zhong et al. carried out a systematic review and meta-analysis in 2023 [112], with the aim being to investigate the statistical association between CS and the development of asthma in the offspring. The results showed that children and adolescents born via CS were at an increased risk of developing asthma as compared to those born via VD. Subgroup analyses showed that the relationship between CS and asthma was not modified by the CS type, asthma type, or cohort design. However, the increased incidence of asthma in children and adolescents born via CS may be influenced by sex and geographical region. Other authors, such as Lee et al., concluded that the combination of prenatal antibiotic exposure and CS might be potentially associated with small-airway dysfunction in preschool-age children, favoring asthma inception in children through changes in the gut microbiome early in life [113].

The abovementioned discrepancies warrant further research into this issue, considering all possible confounding factors, such as infant diet; perinatal factors (a younger maternal age and assisted reproductive technology); and a parental factor (a parent with asthma) that may play a role in the development of asthma in offspring.

**Table 2:** The effects of the mode of delivery on children’s health outcomes, particularly focusing on microbiota diversity, immune responses, and the development of conditions like asthma and atopic dermatitis: description of the studies.

References		Year		Type of Study		Study Population		Objective		Methods		Summary of Major Findings
Lee et al. [113]		2023		Retrospetive cohort study		213,661 mother–child dyads in Manitoba, Canada		Examine the combined effect of prenatal antibiotic exposure and delivery mode on childhood asthma inception.		Utilized Manitoba’s health administrative data to analyze maternal antibiotic prescriptions and child asthma incidence.		Prenatal antibiotic exposure and cesarean delivery were associated with a higher risk of childhood asthma. The combined exposure intensified this risk.
Boker et al. [109]		2019		Case–control study		509 children aged 2–14 years—257 were asthmatic children and 252 control group		To investigate the link between cesarean section and bronchial asthma development.		Review of health records and parental questionnaires.		Cesarean section is associated with a higher risk of developing bronchial asthma in children.
Adeyeye et al. [111]		2017		Longitudinal cohort study		6157 infants and 5034 mothers in New York		Assess the effect of cesarean delivery on the risk of developing wheezing and food allergies in early childhood.		Data from the Upstate KIDS study; used modified Poisson regression and multinomial logistic regression.		Infants delivered via cesarean section had a higher risk of wheezing and food allergies; breastfeeding did not fully mediate this association.
Chu et al. [108]		2017		Case–control study		573 cases and 812 controls		Evaluate the association between cesarean section without medical indication and the risk of childhood asthma, and the effect of exclusive breastfeeding.		Unconditional logistic regression models in SAS.		Cesarean section without medical indication was associated with a significantly increased risk of childhood asthma (adjusted OR = 1.58). This risk was attenuated in children exclusively breastfed for the first six months (adjusted OR = 1.39).
Jakobsson et al. [110]		2014		Cohort study		24 infants (15 vaginally delivered, 9 by C-section)		Investigate how the mode of delivery affects microbiota development in infants and its relation to Th1/Th2 immune responses.		Characterized intestinal microbiota using pyrosequencing of 16S rRNA genes at multiple time points (1 week, 1, 3, 6, 12, and 24 months). Measured venous blood levels of Th1- and Th2-associated chemokines at 6, 12, and 24 months.		Infants delivered by C-section had lower microbiota diversity, delayed Bacteroidetes colonization, and reduced Th1 responses during the first 2 years of life.
Penders et al. [104]		2013		Prospective cohort study		1032 infants from the Netherlands and Germany		Investigate the role of early intestinal microbiota in the development of atopic dermatitis (AD).		Fecal samples were collected at 1 week, 1, 4, 12, and 26 weeks and analyzed using real-time PCR and sequencing.		Early colonization with *Clostridium difficile* and reduced diversity of Bacteroides and Bifidobacteria were associated with an increased risk of AD in infants.

### 4.3. Infant Diet

Following birth, breastfeeding moderates the development of infant immune system. As Waidyatillake et al. asserted in their review [114], breastfeeding favors lung growth and improves lung function. Table 3 provides a comprehensive overview of the eight scientific articles on the relationship between breastfeeding, infant feeding modes, the gastrointestinal microbiome, and asthma risk in children, including different methodological approaches and study-specific findings.

Ogbuanu et al. [115] showed that breastfed children have increased lung volumes by the age of 10 years and hypothesized that it is due to the mechanical stimulus associated with breast suckling. Further evidence that breastfeeding is beneficial for lung function is provided in the aforementioned review by Waidyatillake et al. [114] and Miliku et al. [116], which may be due to reduced infections and increased height in breastfed infants. 

Furthermore, breastfeeding may be related to adequate respiratory health through several potential mechanisms, including modulation of gut microbiota, epigenetics, immunity, and lung development [117]. Breast milk contains a wide variety of bioactive factors that support the development and maturation of the infant gut, such as complex mixture of Human Milk Oligosaccharides (HMOs), lactoferrin, lysozyme, immunoglobulins, and lipids [118]. These elements contribute to produce an intestinal microbiome clearly different from that of the one in formula-fed infants [119]. The diverse and personalized prebiotic and probiotic components of human milk are not present in infant formulas and, therefore, cannot optimally contribute to the natural development of the infant gut microbiota. This may lead to the impaired development of the immune system, together with increased predisposition to asthma in adulthood [43]. In addition, one of the main cytokines in breast milk, the transforming growth factor β (TGF-β), is involved in maintaining intestinal homeostasis, regulating inflammation, and developing oral tolerance [118,120]. Another important issue related to breastfeeding is the skin-to-skin contact that occurs between the mother and the infant during the process which may provide an additional source of protective maternal microbes to the nursing infant [121]. 

Breast milk has a complex microbial composition with a high microbial diversity and large individual differences. Most of the microbiota in breast milk belongs to Proteobacteria and Firmicutes and is mainly *Streptococcus* and *Staphylococcus* [122]. Moreover, typical bacteria from the oral and skin environments are also found in breast milk [123,124]. Wang et al., in 2023 [122], found that the most abundant bacteria in breast milk were *Acinetobacter*, *Stenotrophomonas*, *Sphingopyxis*, *Pseudomonas*, and *Streptococcus*, whereas the most abundant bacteria in infant fecal samples were *Bifidobacterium*, *Klebsiella*, *Streptococcus*, Bacteroides, *Escherichia*–*Shigella*, and *Lactobacillus*. Of all of them, *Acinetobacter*, *Bifidobacterium*, *Klebsiella*, and *Lactobacillus* existed in both human milk and infant feces. Breast milk microbiota help to produce beneficial gut microbiota in infants, including increased colonization by Bifidobacteria and reduced abundance of *C. difficile* and *Escherichia coli* [125]. 

Breast milk microbes and other factors in human milk influence the development of the infant immune system, enhance resistance against infection, and protect against the development of allergies and asthma later in childhood [126,127,128]. In a meta-analysis of 117 studies, Dogaru et al., in 2014 [129], found that breastfeeding was associated with a 22% reduced risk of asthma and noticed that the greatest effect was observed during early childhood. 

As mentioned above, HMOs are the main factor that favors the growth of Bacteroides, *Bifidobacterium* and *Lactobacillus*, which are fermenting bacteria, and, therefore, can increase the levels of gastrointestinal and circulating SCFAs in the infant [130]. Also, due to their prebiotic character, the HMOs from breast milk have been attributed beneficial effects that may include reinforcement of the immune system with better immune response to infective agents; improved resistance to infections of the gut; immunomodulation against food allergies, asthma, and atopic dermatitis; and, finally, decreased risk of chronic diseases. HMOs can modulate the balance between Th1/Th2 immunity and provide essential nutrients for brain development and cognition [131]. Moreover, higher proportions of certain HMOs, specifically 1-2-fucosyltransferase (FUT2)-dependent oligosaccharides (namely 2′-fucosyllactose), have been associated with a decreased risk of respiratory disease in infants [132,133]. 

Other important human milk factors are antibodies (including IgG and IgA), which provide passive immunity to the infant. Immune cells, such as neutrophils and macrophages, and cytokines are also present in breast milk, along with bactericidal enzymes and antiviral factors [118,134]. Nutrients and growth factors in breast milk have been shown to regulate innate immunity, while fatty acids such as PUFAs can modulate neonatal cytokine responses [120]. The content of PUFAs, such as n-6 and n-3, in breast milk and its impact on the immune system has been particularly well studied: n-3 PUFAs are anti-inflammatory and protect the intestinal barrier [135]. Lower amounts of n-3 fatty acids in breast milk are associated with a higher risk of atopy in infants [136].

Despite the many protective factors transmitted by breast milk, the debate remains open as to whether breastfeeding is protective against childhood asthma and to what is their impact on lung function [129,137]. Several studies have shown that asthma risk is reduced in breastfed infants [137,138,139,140,141,142,143]. More specifically, in the CHILD study, the authors observed that breastfeeding is associated with lower rates of wheezing in the first year of life [141] and lower odds of possible or probable asthma by three years of age [143]. This is consistent with other cohorts in Canada, Sweden, the United States, and Australia. However, other studies claim that there is no association or even an increased risk of asthma in breastfed children.

Rosas-Salazar et al., in 2022 [144], examined, in a prospective study of 1456 healthy infants enrolled in a population-based birth cohort, the interactions between the mode of delivery and breastfeeding on gut microbiomes in infancy and the development of recurrent wheeze in infants. They showed that there was a significant interaction between the mode of delivery and breastfeeding on recurrent wheeze. In VDIs, breastfeeding decreased the odds of recurrent wheeze by ~40%; however, in CSDIs, there was no effect of breastfeeding on recurrent wheeze. These results demonstrate that the protective effect of breastfeeding on the development of recurrent wheeze is only seen in children born by vaginal delivery, and these findings could explain the discrepant results of prior studies. Bigman et al., in 2020 [145], in a longitudinal study using data from 1177 mother–infant pairs who took part in the Infant Feeding Practices Study II in 2005–2007 and the Year 6 Follow-Up Study, in 2012, provided evidence that exclusive breastfeeding for the first 3 months may reduce the risk of respiratory allergies and asthma in children at 6 years of age, but concerning asthma, statistical significance was reached only in children without a family predisposition to asthma.

As Miliku et al. reviewed in 2018 [116], infant feeding can be described and measured in many ways, making it difficult to compare results between studies and causing confusion. To describe exclusive, complete, predominant, partial, and mixed breastfeeding, different terminologies and criteria are applied inconsistently. WHO [146] defines exclusive breastfeeding as feeding human milk only (including donor human milk), without any food, water, or other fluids, although vitamin and mineral supplements or medicine syrups are allowed. Most studies do not capture enough information to apply the definition described by WHO, and many do not document the total duration of breastfeeding (age of the infant at weaning); therefore, systematic reviews have been limited to comparing breastfeeding in the terms “ever versus never” or breastfeeding “more versus less” [129,137]. As a result, it is not possible to correctly assess the possible “dose effect” useful to infer causality [116]. 

Today, breastfeeding has evolved, and there are other factors related to it that may be potentially important and are often ignored in epidemiological studies, for example, the method of feeding. Breast milk can be used directly or previously expressed, bagged (in some cases frozen and thawed), and supplied with a bottle. It is also important in mixed breastfeeding to consider the type of complementary feeding, since it can be infant formulas, solid food, or semi-solid food. The relative proportion of breast milk taken by the infant compared to other nutritional sources is also important. Finally, it is essential to consider whether the supplied breast milk is from the mother or from donors because, in milk banks, breast milk is pasteurized to ensure its sanitary quality. There are studies that have shown how its protective effect against wheezing during childhood is decreased due to the supplementation with infant formulas. This effect was not observed when supplementation was performed with solid foods [141]. 

On the other hand, several authors have reported that breastfeeding directly by suckling appears to be more protective than bottle-feeding with human milk [143,147]. A recent study carried out within the CHILD cohort reported a dose-dependent reduced risk of wheezing [141] and asthma [143] among breastfed children. This reduction was lower in infants fed with pumped breast milk as compared to those directly breastfed; however, the reduction was higher in both groups of infants as compared the group fed infant formula [143]. One of the potential explanations for this difference is the possible alteration of the bioactive components of human milk during the extraction, refrigerated storing, freezing, and thawing [148,149]. Another possibility is the transfer to breast milk of asthmogenic contaminants from breast pumps or storage containers, such as bisphenol A, present in plastics [150,151,152]. As already said, breastfeeding provides skin-to-skin contact between the mother and the infant, being a source of exposure to protective microorganisms present on the breast skin, nipple, and maternal halo. Lastly, the suckling exercise at the breast by the infant can stimulate lung growth due to the effort that must be made to obtain food [114].

The current available scientific evidence supports the use of donated human milk (DHM) for very premature or sick newborns when their own mother’s milk is not available or is insufficient [153,154]. The large increase in the use of pasteurized DHM is evident but is rarely reported or studied in relation to respiratory health. This is relevant because DHM is submitted to pasteurization to ensure its microbiological safety in human milk banks, but this treatment affects some of its bioactive compounds, reducing, for instance, the concentrations and biological activities of all Igs classes. Among Igs, IgA is the most abundant in human milk, and its concentration is reduced by 20–60% after pasteurization [155]. These findings emphasize that the mode of feeding (exclusive breastfeeding, with formula, donated milk, etc.) is a very important factor to consider when investigating the effects of human milk on asthma and allergy [116]. 

**Table 3:** The effects of breastfeeding on children’s health outcomes, particularly focusing on microbiota diversity, immune responses, and the development of conditions like asthma and atopic dermatitis: description of the studies.

References		Year		Type of Study		Study Population		Objective		Methods		Summary of Major Findings
Rosas-Salazar et al. [144]		2022		Prospective cohort		1495 infants		To examine interactions between mode of delivery, breastfeeding, infant respiratory and gut microbiomes, and recurrent childhood wheeze.		Analysis of data on respiratory and gut microbiomes.		Exclusive breastfeeding is associated with beneficial microbiome changes and improved immune responses, reducing respiratory illness risk.
Bigman [145]		2020		Observational cohort study		1177 mother–infant pairs		To assess the association between exclusive breastfeeding for the first 3 months of life and respiratory allergies and asthma at age 6 years.		Analysis of cohort data (including breastfeeding status and respiratory health assessments at age 6 years) from participants who took part in the Infant Feeding Practices Study II and the Year 6 Follow-Up Study.		Exclusive breastfeeding for the first 3 months of life was associated with a reduced risk of respiratory allergies and some forms of asthma in children by age 6.
Abarca et al. [139]		2019		Observational		15,642 children aged 3 to 5 years.		To investigate the relationship between supplemental breastfeeding and asthma prevalence.		Analysis of cohort data with adjustment for adverse childhood experiences		Found a significant association between longer breastfeeding duration and reduced asthma prevalence in children exposed to adverse childhood experiences.
Ahmadizar et al. [140]		2017		Longitudinal cohort study		960 children (aged 4–12 years) using regular asthma medication.		To examine the association between breastfeeding and the risk of asthma exacerbations later in childhood.		Analysis of survey data of PACMAN study.		Breastfeeding is associated with a decreased risk of asthma exacerbations in later childhood.
Azad et al. [141]		2017		Longitudinal birth cohort study		2773 infants at one year of age.		To examine the impact of breastfeeding and maternal asthma on wheezing in the first year of life.		Analysis of survey data from the Canadian Healthy Infant Longitudinal Development (CHILD) birth cohort.		Found that breastfeeding reduced the risk of wheezing in a dose-dependent manner among infants born to mothers with asthma.
Klopp et al. [143]		2017		Prospective cohort study		3296 infants		To determine the association of different modes of infant feeding with the risk of childhood asthma.		Analysis of feeding modes and asthma development within the CHILD cohort study.		Any mode of feeding that included breast milk was associated with a decreased risk of asthma at 3 years compared to feeding modes that did not include breast milk.
den Dekker et al. [142]		2016		Prospective cohort study		5675 children		To investigate the association between breastfeeding and asthma outcomes at 6 years old.		Analysis of breastfeeding duration and asthma symptoms using questionnaires and medical records embedded in the Generation R Study.		Longer breastfeeding duration was associated with a reduced risk of asthma symptoms. The protective effect was more pronounced in children with non-atopic mothers.

### 4.4. Antibiotics

Epidemiological studies in humans indicate that broad-spectrum antibiotic exposure may play a role in the development of asthma and atopy. Table 4 summarizes the most significant observational studies exploring the relationship between antibiotic exposure during gestation and in childhood and the risk of developing asthma, providing an overview of the objectives, methods, and main findings of each investigation.

#### 4.4.1. Prenatal Exposure to Antibiotics

During the last decade, different epidemiological studies have reported a link between allergic disease and antibiotic exposure during the prenatal period [156,157,158,159]. It has been repeatedly shown that prenatal exposition to antibiotics is associated with the occurrence of asthma during childhood [113,156,159,160,161] (Table 4). 

One prospective study carried out by Stensballe et al. in 2013 on a birth cohort [159] showed increased risk of asthma exacerbations if mothers had used antibiotics during the third trimester of pregnancy. Also, they replicated these findings in an unselected national birth cohort, finding also increased use of inhaled corticosteroids in children of mothers who consumed antibiotics at any time during pregnancy. This increased risk of asthma was also observed in the subgroup of mothers using antibiotics for non-respiratory infections. Metsala et al., in 2015 [157], also observed this effect and, after a stratified analysis per antibiotic used, found that the strongest association was with cephalosporins. Mulder et al. [158] conducted a study in which children with asthma (n = 1228) were compared to siblings without asthma. They concluded that antibiotic use in the third trimester of pregnancy was associated with a small increase in the risk of asthma at preschool age (both the case–sibling and case–control analysis) and that this association was robust to time-invariant confounding or exposure-time trends. Moreover, Chu et al. [156] observed that the maternal use of penicillin or chloramphenicol is associated with later infant asthma. In 2019, Lijun Bai et al. [162] conducted a meta-analysis to evaluate the risk of the use of antibiotics in each of the three pregnancy trimesters on childhood asthma or wheeze. They found a positive association between prenatal antibiotic use in every trimester and childhood asthma or wheeze. However, adjustment for confounders such as infections during pregnancy and siblings decreased the relative risk estimates, supporting the concept that these associations are, at least in part, due to confounding factors. The current available research probably does not take sufficiently into account confounding factors such as maternal asthma history, smoking, mode of delivery, birth weight, or even breastfeeding. Additionally, as Milliken et al. reviewed [163], in most studies, there is no control of the number of antibiotic courses administered, the indication for antibiotics, the use of broad-spectrum or narrow-range antibiotics, the timing of use, the dose-dependent nature of the effect, the class of antibiotics used, or if the mother suffered the same condition [160,164].

#### 4.4.2. Antibiotic Exposure in Early Life

In a systematic review and meta-analysis carried out in 2022 by Duong et al. [165] which included 51 studies linking the use of antibiotics in the first five years of life and the development of asthma, the authors concluded that antibiotic exposure was associated with increased risk of developing asthma. Marra and colleagues, in 2009 [166], found that antibiotic exposure in the first year of life was associated with a slight increase in the risk of developing asthma in early childhood after adjusting for a great number of confounders. Also, they concluded that, as the number of courses of antibiotics increased, the asthma risk increased, with the highest risk being in children who received more than four courses. Finally, all antibiotics were associated with an increased risk of developing asthma, except for sulfonamides. Muc et al., in 2013 [167], conducted a questionnaire-based study and found that antibiotic exposure in the first year of life plays a significant role in the development of asthma and allergic rhinitis in children. Additionally, Hoskin-Parr et al. [168], assessing data from 4952 children enrolled in the Avon Longitudinal Study of Parents and Children, found a dose-dependent association between antibiotic usage during the first 2 years of life and asthma at 7.5 years of age. H et al., in 2019 [169], used administrative health data from a total of 2644 children from the CHILD cohort to examine the association between antibiotic prescriptions (age <1 year) and asthma incidence (ages 1–4 years) and concluded that antibiotic exposure in the first year of life increases the risk of being diagnosed with astma in childhood. This risk was reduced with increased gut microbiota α-diversity, suggesting that gut microbiota, represented by diversity and differentially expressed taxa, might be a significant mediator between antibiotics and asthma. The use of antibiotics probably disturbs the balance of microflora skewing the developing immune system towards Th2.

For everything stated throughout this review, more research is needed to better understand the complex interplay of these factors in determining the risk of asthma in children. Detailed investigations are still needed to better understand the relationship between various factors previously addressed, the composition of the gut and lung microbiota, and their impact on the immune response. Most of the evidence comes from experimental studies, and clinical evidence remains limited. Therefore, the future of research in this field is broad and promising. There is a need for well-designed, randomized, controlled clinical trials that clearly define the target population, the variables to be measured, and the strategies for data analysis and interpretation—more specifically, population-based studies—focused on age groups with a high incidence of asthma.

- Research focused on well-defined phenotypes or pathogeneses as dependent variables.
- Metagenomic and metabolomic analyses of the gut–lung axis and its relationship with various parameters of the immune system, especially those related to bronchial inflammation.

Data analysis strategies that consider the numerous factors that influence the composition of the microbiota, requiring a detailed description of these factors, which could act as independent variables.

The use of the scientific evidence gathered to date on the mechanisms and metabolites involved in the microbiota–gut–lung axis opens the door to the possibility of designing therapies based on it. The modulation of the host’s physiological functions by the microbiota is a highly relevant therapeutic option, and all research efforts in this direction are therefore necessary.

**Table 4:** The effects of antibiotic exposure during gestation and in childhood and the risk of developing asthma.

**Prenatal antibiotic**												
**References**		**Year**		**Type of study**		**Study population**		**Objective**		**Methods**		**Summary of major findings**
Turi et al. [160]		2020		Retrospective cohort study		84,214 mother–child dyads.		Assess timing and spectrum of prenatal antibiotic exposure and risk of childhood asthma.		Analysis of electronic health records from Tennessee Medicaid Program (TennCare).		Higher cumulative doses and broader spectrums of prenatal antibiotic exposure were associated with an increased risk of childhood asthma.
Mulder et al. [158]		2016		Retrospective cohort study		1228 children with asthma were compared to 1228 siblings without asthma.		To investigate the association between antibiotic use during pregnancy and asthma in preschool children, considering potential confounding factors.		Retrospective cohort analysis using healthcare databases.		Antibiotic use in the third trimester of pregnancy was associated with a small increased risk of asthma in preschool children. This association was robust to non-time-varying confounders or temporal trends in exposure, reinforcing the important role of the gut microbiota early in life in the development of childhood asthma.
Chu et al. [156]		2015		Cohort study		39,907 children		To investigate the relationship between periconceptional and gestational exposure to antibiotics and the development of childhood asthma.		Cohort data analysis from Collaborative Perinatal Project.		Periconceptional and gestational exposure to antibiotics was associated with an increased risk of developing childhood asthma.
Metsälä et al. [157]		2015		Population-based cohort study		6690 case–control child pairs at 3 years of age.		To examine the association between prenatal and post-natal exposure to antibiotics and the risk of asthma in childhood.		Study using national registry data.		Both prenatal and post-natal exposure to antibiotics were associated with an increased risk of childhood asthma.
Stensballe et al. [159]		2013		Cohort study		411 infants born of mothers with asthma and 30,675 infants as replication were included followed prospectively for 5 years.		To assess the association between antibiotic use during pregnancy and the risk of asthma in early childhood.		Analysis using healthcare databases from Copenhagen Prospective Study on Asthma in Childhood (COPSAC) and Danish National Birth Cohort (DNBC), with detailed maternal antibiotic use data and asthma assessments in early childhood.		Antibiotic use during pregnancy was associated with an increased risk of asthma in early childhood, supporting a role for bacterial ecology in pre- or perinatal life for the development of asthma.
**Antibiotic during early life**												
**References**		**Year**		**Type of study**		**Study population**		**Objective**		**Methods**		**Summary of major findings**
Muc et al. [167]		2013		Cross-sectional study		1063 children		To assess the association between exposure to paracetamol and antibiotics in early life and the risk of asthma in childhood.		ISAAC-based environmental and core asthma and rhinitis questionnaires.		Exposure to paracetamol and antibiotics in early life was associated with an elevated risk of asthma in childhood.
Hoskin-Parr et al. [168]		2013		Prospective cohort study		4952 children		To investigate the dose-dependent relationship between antibiotic exposure in the first two years of life and the development of asthma and other allergic diseases by 7.5 years.		Analysis of Avon Longitudinal Study of Parents and Children (ALSPAC) data.		There was a dose-dependent relationship between antibiotic exposure in the first two years of life and increased risk of asthma and other allergic diseases by 7.5 years, but it did not appear to be mediated through an association with atopy.
Marra et al. [166]		2009		Population-based cohort study		251,817 infants at 1 year of life.		To investigate the association between antibiotic use in children and the risk of developing asthma.		Using administrative data, birth cohorts from 1997 to 2003. Antibiotic exposure was determined for the first year of life. After the first 24 months of life, the incidence of asthma was determined in both those exposed and not exposed to antibiotics in the first 12 months of life.		Antibiotic use in children was associated with an increased risk of asthma, and this risk increases with the number of courses of antibiotics prescribed.

## 5. Summary

The new concept “gut–lung axis” aims to describe how microbes in the gut may impact immune function in the lungs. This interaction hinges on the relationship between intestinal microbiota and pattern-recognition receptors of the innate immune system, particularly TLRs. Activation of TLR signaling by microbial patterns regulates inflammation and innate immune responses. Dendritic cells serve as intermediaries in the communication between gut microbiota and immune cells. They detect microbial patterns, promote immune tolerance in the intestine, induce phenotypic changes, and migrate to lymph nodes, influencing T-cell preparation and migration to other body parts, including the respiratory mucosa.

Also, microbial-derived metabolites, such as short-chain fatty acids (SCFAs), influence gene expression, cytokine production, cell differentiation, proliferation, and apoptosis. SCFAs, acting as histone deacetylase (HDAC) inhibitors, foster a tolerogenic, anti-inflammatory cell phenotype, particularly in regulatory T cells (Tregs). In asthma models, SCFAs like propionate and butyrate induce FoxP3, potentially reducing allergic airway inflammation. Maternal intake of acetate during pregnancy is associated with a decreased risk of allergic airway disease in offspring.

Studies in animal models emphasize the “early life critical window” to understand the gut–lung axis. Changes in intestinal microbial compositions during early life are linked to asthma and allergy. Studies manipulating the intestinal microbiome with antibiotics demonstrate the increased severity of those diseases. Additionally, longitudinal studies in humans reveal differences in intestinal microbiota profiles between infants who develop asthma and those who do not. It is critical to identify microbial dysbiosis during the first 100 days of life. 

Dysbiosis is characterized by specific changes in bacterial taxonomies at the family, genus, and species levels. Factors such as diet during pregnancy can modify the intestinal microbiota profile and impact the risk of asthma in later stages of life. Past and recent research, particularly focused on adherence to a Mediterranean diet (MD), highlights its protective effects. 

The complexity of the relationship between maternal dietary factors, specific nutrients, and the risk of asthma and allergy in the offspring requires a deep understanding of the various influencing factors.

With respect to the mode of delivery, the findings are conflicting. Studies indicate that infants born through CS delivery may experience lower microbiota diversity, reduced abundance of specific microbial phyla, and altered levels of immune system-related chemokines. Some studies associate emergency CS with an elevated risk of wheeze and food allergy. However, the relationship between CS and asthma may be influenced by factors such as programmed vs. emergency CS, sex, and region. The roles of potential confounders like mother and infant diet, perinatal factors, parental factors, maternal age, or assisted reproductive technology are to be established. 

Epidemiological studies over the past decade consistently suggest a link between prenatal exposure to antibiotics and the subsequent development of allergic diseases, particularly asthma, during childhood. Broad-spectrum antibiotic exposure during the first five years of life has been implicated in the development of asthma and atopy. Multiple studies consistently demonstrate that antibiotic exposure in the first year of life is linked to an elevated risk of developing asthma in a dose-dependent manner. The disturbance of the gut microbiota balance is thought to be a significant mediator between antibiotics and asthma, impacting the developing immune system and predisposing individuals to Th2 immune response.

However, insufficient attention is probably given to confounding factors such as maternal history of asthma, smoking, mode of delivery, birth weight, and breastfeeding. Not only exposure but also timing, type, and dose are of importance. For instance, cephalosporins show the strongest association with future asthma, and the third trimester seems to be the most sensitive to those drugs. 

In conclusion, it is crucial to investigate the relationship between microbial dysbiosis and the early onset of asthma to determine if it is a cause or merely an associated finding. Specific mechanisms of how microbial dysbiosis in the gut–lung axis affects health and causes asthma should be described, identifying the relevant microbes and the role of specific antibiotic therapy.

Other areas of interest include the impact of viral infection and/or fungal dysbiosis on immune homeostasis and the effect of dietary intervention on microbial dysbiosis and asthma development.

Finally, the role of probiotics needs further investigation. Studies have shown positive results in children with allergic asthma when different probiotics are administered for short periods. However, more research is needed not only in patients with asthma but also in those at risk of asthma and microbial dysbiosis to clarify their preventive effect, their ability to modulate epithelial barrier function, and their interaction with the immune system.
